# Comments on the Eslicarbazepine Acetate Section of the Article ‘Therapeutic Drug Monitoring of the Newer Anti-Epilepsy Medications’

**DOI:** 10.3390/ph3123629

**Published:** 2010-12-17

**Authors:** Ahmet H. Öztiryaki, Patricio Soares-da-Silva

**Affiliations:** BIAL - Portela & C^a^, S.A., S. Mamede do Coronado, Portugal

**Keywords:** anticonvulsants, anti-epileptic drugs, drug monitoring, drug toxicity, epilepsy, seizures, carbamazepine, oxcarbazepine, eslicarbazepine acetate

## Abstract

The recent review of Matthew D. Krasowski on ‘Therapeutic Drug Monitoring of the Newer Anti-Epilepsy Medications’ is a useful foundation of comparative interpretations on our current knowledge about therapeutic drug monitoring. Within the review, the statement that therapeutic drug monitoring has a minimal role in the therapeutic use of eslicarbazepine acetate due to its relatively predictable pharmacokinetics reflects the existing body of evidence although some information such as eslicarbazepine acetate’s chemical structure, proportions of its metabolites, their pharmacokinetics and chiral method of plasma level measurement need to be revised. These critical characteristics differentiate the novel compound from former dibenzazepines such as carbamazepine and oxcarbazepine in its clinical effects and needs for therapeutic drug monitoring.

## The Rationale behind this Communication

We read with interest the recent review of Krasowski on ‘Therapeutic Drug Monitoring of the Newer Anti-Epilepsy Medications’, published in the June 2010 edition of *Pharmaceuticals* [1]. The evidence-based information about relevant circumstances which may require therapeutic drug monitoring (TDM) is founded on useful interpretations of the current knowledge in this area. The section related to eslicarbazepine acetate (ESL) was of special interest to us as the development of this antiepileptic compound took place in the laboratories of BIAL - Portela & C^a^ S.A. located in São Mamede do Coronado, Portugal.

## The Review Provides Useful Information Related to Anti-Epileptic Drugs (AEDs)

The comparative information depicted was useful in terms of providing comprehensive knowledge on TDM needs for AEDs in general, and very much in line with the data published in peer reviewed journals, non-published data on file in our records, and also the publicly available ESL (Zebinix^®^) summary of product characteristics (SPC) approved by the European Medicinal Agency (EMA) [2,3]. The statement that TDM has a minimal role in the therapeutic use of ESL due to its relatively predictable pharmacokinetics was also in compliance with the pharmacokinetic and pharmacodynamic data gathered from early preclinical and initial Phase I through late Phase III clinical trials.

## The Referred Chapter Provides Additional Room for Discussion on the Particular Characteristics of Eslicarbazepine Acetate

### The Chemical Structure of Each Dibenzazepine Is Unique and Different, Substantiating the Differences in Their Plasma Kinetics and Clinical Effects

**Figure 1:**
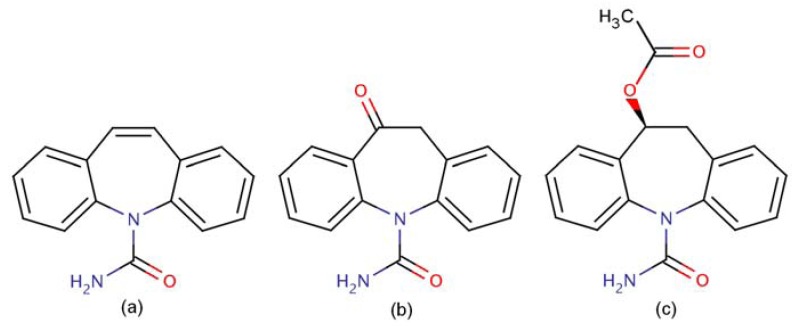
The chemical structures of three different dibenzazepines: **(a)** carbamazepine (CBZ); **(b)** oxcarbazepine (OXC) and **(c)** eslicarbazepine acetate (ESL).

However, some of the ESL related information in the tables and figures were not in agreement with the referred text on page 1915, nor did the chemical structure in ‘Figure 1’ of the same page precisely represent the compound. In fact, the correct structure of ESL differs from that of other dibenzazepines in an acetate group that requires the addition of a methyl group to the existing drawing [2]. As seen on Figure 1 below, there are no double bonds in the common azepine rings of the dibenzazepines except in carbamazepine.

### Eslicarbazepine Acetate and Its Main Active Metabolites Differ from CBZ by Not Auto-inducing Its Metabolism

An additional note in the same column of the ‘Table 2’ highlights the propensity of auto-induction with chronic dosing as a characteristic of ESL that favors the use of TDM. However, this highlight in ‘Table 2’ is not in line with our current knowledge that indicates ESL as a compound not susceptible to cause metabolic autoinduction, which was also defined paradoxically within the text of the same review on p. 1915 [4].

### Some Critical Pharmacokinetic and Safety Related Information Seem To Be Incorrect Despite the Accuracy of the Text

According to the SPC and published research, ESL displays a bioavailability exceeding 90% and a time to peak concentration (t_max_) of 2-3 hours, which is not in line with the data presented in ‘Table 1’ on page 1913 [3,4]. Apart from that, in contrast to the information in the first line of the ‘Table 2’ on page 1914, the pharmacokinetics of ESL are not affected by mild to moderate hepatic impairment as an important point that was also addressed within the text of the review, as well as the SPC [3,5]. 

### The Concerns on Interpretation of TDM Was Based on a Misinterpretation of the Different Pharmacokinetic Profile of ESL Compared to OXC

In addition to the conflicting information in the ‘Table 2’, the third column also indicates OXC as an active metabolite of ESL, which could potentially complicate the interpretation of TDM. However, as emphasized within the text of the review and referring to published literature as well as the SPC, OXC is a marginally produced minor metabolite comprising less than 1% of the total ESL analytes in the human plasma [4]. On the contrary, OXC, which undergoes major conversion to eslicarbazepine and *R*-licarbazepine in a 4/1 ratio, was shown to generate a steady-state proportion of 3.5% of OXC in plasma, which is more than 3-fold higher compared to that of ESL [6]. Hence, this significant difference indicates that such a limiting factor within the ‘Table 2’ should be clearly highlighted for OXC itself instead of ESL, as this might be considered to cause a relevant interference with TDM.

### The Model Recommended To Detect ESL and Its Metabolites Is a More Complicated Quadrupole HPLC-MS Method

The fact given within the review that high-performance liquid chromatography-ultraviolet (HPLC-UV) has been developed as an enantioselective method for the detection of ESL and its main active metabolites such as eslicarbazepine is true [7]. As additional information, a more sophisticated technique such as quadrupole HPLC-mass spectrometry (HPLC-MS) has also been described elsewhere in detail as the standard method recommended and used during the clinical development program of ESL in humans [8,9,10]. Although simple and cost-effective measurements of the mono-hydroxy derivative (MHD) levels would be sufficient for clinical monitorization needs, the quadrupole HPLC-MS method proved itself as the most sensitive and accurate chiral method available for the detection of ESL and its metabolites simultaneously for purely scientific purposes.

## Summary

We believe that with the inclusion of some revisions and additional information, this comprehensive review will provide the clinicians with accurate guidance and useful hints on therapeutic plasma level monitoring of antiepileptic compounds, and will close some of the information gaps in this field.
